# Evaluating the Acceptability and Utility of a Personalized Wellness App (Aspire2B) Using AI-Enabled Digital Biomarkers: Engagement Enhancement Pilot Study

**DOI:** 10.2196/63471

**Published:** 2025-05-14

**Authors:** Calissa J Leslie-Miller, Shellen R Goltz, Pamela L Barrios, Christopher C Cushing, Teena Badshah, Corey T Ungaro, Shankang Qu, Yulia Berezhnaya, Tristin D Brisbois

**Affiliations:** 1 Department of Clinical Child Psychology, University of Kansas, Lawrence, KS, United States; 2 Life Sciences, Global R&D, PepsiCo Inc, 700 Anderson Hill Rd, Purchase, NY, 10577, United States; 3 Life Span Institute, University of Kansas, Lawrence, Kansas, United States; 4 Data Science and Analytics, R&D, PepsiCo Inc, Valhalla, NY, United States

**Keywords:** wellness application, development protocol, AI-enabled digital biomarkers, acceptability, nutrition, sleep, fitness, mobile phone

## Abstract

**Background:**

There is significant global interest in promoting wellness, with digital solutions like mobile health apps being broadly downloaded; yet, there is a challenge in maintaining engagement for long-term behavior change. Developing a widely accepted mobile wellness app is imperative for advancing personalized wellness interventions.

**Objective:**

The primary objective of this study was to evaluate the Aspire2B wellness app (powered by Salus Optima), designed to exceed industry standards for participant engagement by incorporating evidence-based behavior change strategies and to assess its acceptability (eg, liking the face scan) and utility (eg, willing to use the face scan technology for other health insights) as a digital health solution.

**Methods:**

Participants aged 18-65 years, who were smartphone and fitness tracker users, were recruited in the United States during March-May 2022. Participants received US $5 compensation for downloading the app, with no further incentive for usage. Following completion of onboarding (ie, survey questions about lifestyle behaviors), participants were placed in either a nutrition, sleep, or fitness 4-week challenge. During the challenge, participants used various app features at their own will, such as a facial scan for wellness insights (eg, heart rate and biological age), recipes, and workout videos. These interactions with the app were cumulatively evaluated as engagement metrics. Participants were also asked to answer offboarding questions to evaluate any changes to lifestyle behaviors and experience using the app features (eg, acceptability of face scan experience).

**Results:**

Out of the 398 people who created an account, 85.9% (342/398) completed onboarding and a face scan. Following this, 74.9% (298/398) of users completed additional survey questions about current wellness behaviors. Notably, interaction with the app was relatively stable from week 2 to 4 (173/398, 43.5%), outperforming industry standards by roughly 3×. In addition, on average, participants completed 2.1‐2.7 face scans per week, with approximately 7% (24/342) of participants maintaining regular use of face scan technology for 4 weeks. In users who completed offboarding questions, 88.8% (111/125) found Aspire2B credible, 64.8% (81/125) liked the face scan experience, 7.2% (9/125) disliked the face scan experience, and 83.2% (104/125) said they would use face scan technology for other insights into their health.

**Conclusions:**

These findings highlight strong initial engagement with Aspire2B, followed by significant sustained user engagement over a 4-week period. Furthermore, users indicated high levels of credibility and willingness to use face scan technology for wellness insights. These findings collectively demonstrate the capability of a personalized wellness app using AI-enabled digital biomarkers and evidence-supported behavior change techniques to yield positive user perception and provide long-term engagement.

## Introduction

### Background

Around the world, there is substantial and widespread interest in the promotion of wellness. Digital solutions are a common wellness tool for consumers, with over 300 million people using a mobile health app in 2023 [1]. This trend presents an important opportunity for managing population health as there continues to be suboptimal uptake of physical activity, healthy diet, and adequate sleep, which are the most important health behaviors for wellness and longevity [2]. Encouragingly, there exists substantial evidence that basic behavior change strategies can effectively increase the uptake of wellness behaviors [3]. However, it is clear that the majority of wellness app users quickly disengage and stop using the app with novel features providing only a small boost to engagement and retention long-term [3].

Furthermore, for mobile apps to have a meaningful effect on well-being by driving behavior change, it is crucial to pinpoint enduring technologies that will foster engagement over an extended period, allowing sufficient time for behavior shifts to take place [4]. Mobile apps contain 2 classes of features, those that are designed to change behavior and those that are designed to promote engagement. The most common and best supported strategies for behavior change are self-monitoring of performance, tools for facilitating setting effective goals, and feedback mechanisms [5,6]. It is less clear which app features are useful for promoting engagement (trophies, badges, or points within the app), and there is insufficient evidence that they lead to long-term engagement (the field lacks high-quality randomized controlled trials of app engagement strategies). One way to potentially increase engagement is by delivering novel health information using technologies that are intriguing or surprising to users.

Over the last 50 years, a body of evidence has emerged that characterizes the biological factors underpinning the aging process. The notion of biological age is captivating as it offers individuals an estimation of their aging status that transcends chronological age and perceived age [7]. Providing consumers with a biological age that differs from their chronological age has the potential to stimulate behavioral shifts [8]. Specifically, if information reveals that one’s biological age is advancing faster than their chronological age, these individuals may be motivated to adopt behavior changes aimed at bringing the two into closer alignment.

Historically, the science of biological age was limited to high throughput omics research and was mainly accessible to researchers or specialized health care providers [9]. However, it is now possible to estimate an individual’s biological age by using computer vision combined with artificial intelligence models [10]. This advancement brings a cutting-edge scientific concept directly to consumers, who are already familiar with activities such as using their phone’s camera for tasks like facial scans to unlock their iPhone (Apple Inc) [11]. As barriers to accessing personal information about biological age are reduced, the field needs a series of rigorous studies to assess the feasibility and acceptability of apps that leverage the appeal of biological age to drive and maintain engagement in wellness programs, ultimately leading to positive changes in wellness behavior.

### Objectives

Our study aimed to address several key questions. First, we explored who uses the app by examining participants’ characteristics. We also investigated whether users find the Aspire2B app, including its content and features, credible and valuable for providing wellness insights. Another focus was determining whether users remain engaged with the app over time. Finally, we examined which participant characteristics were related to long-term engagement with the app. Given that the app was grounded in behavior change science, we hypothesized that the majority of participants would find Aspire2B credible and enjoyable, use it for wellness insights, and recommend the app to others. Furthermore, we hypothesized Aspire2B retention rates would exceed industry standards, such that more than 20% of users that create an account, complete onboarding, more than 9% interact with the app in week 2, and more than 4% interact with the app in week 4 [12]. In addition, we predicted that participants who were informed of a biological age value higher than their chronological age would be more likely to engage in the app (ie, serving as a nudge or “wake-up” call to engage in more healthful lifestyle behaviors). Finally, we were interested in exploring participant characteristics that would predict higher engagement with the app without a priori predictions, as well as whether improvements in wellness metrics could be detected following program completion.

## Methods

### Participant Recruitment

Participant recruitment was led by Human8, who used one of their partner’s panels to achieve the desired target sample in the United States during March-May 2022. The partner panel consists of willing participants, older than 18 years, who have regular internet access, and have opted in to be part of a database that allows them to voice their opinions with the opportunity to be incentivized. These panelists were invited to complete a recruitment questionnaire to determine eligibility for this study. Additional inclusion criteria required participants to be 18‐65 years old and report that they were smartphone and fitness tracker users. Those who met the inclusion criteria were provided with a link to download the Aspire2B app.

### Ethical Considerations

This study was reviewed and approved by the Sterling institutional review board. Participants were presented information about informed consent, after downloading the app, through a screen that occurred before onboarding. Participants were compensated $5 for downloading the application, with no further incentives for usage (refer to Participant Recruitment section). Images used in this paper do not identify real participants and data analysis was completed on deidentified data.

### Intervention

#### Overview

**Figure 1.**
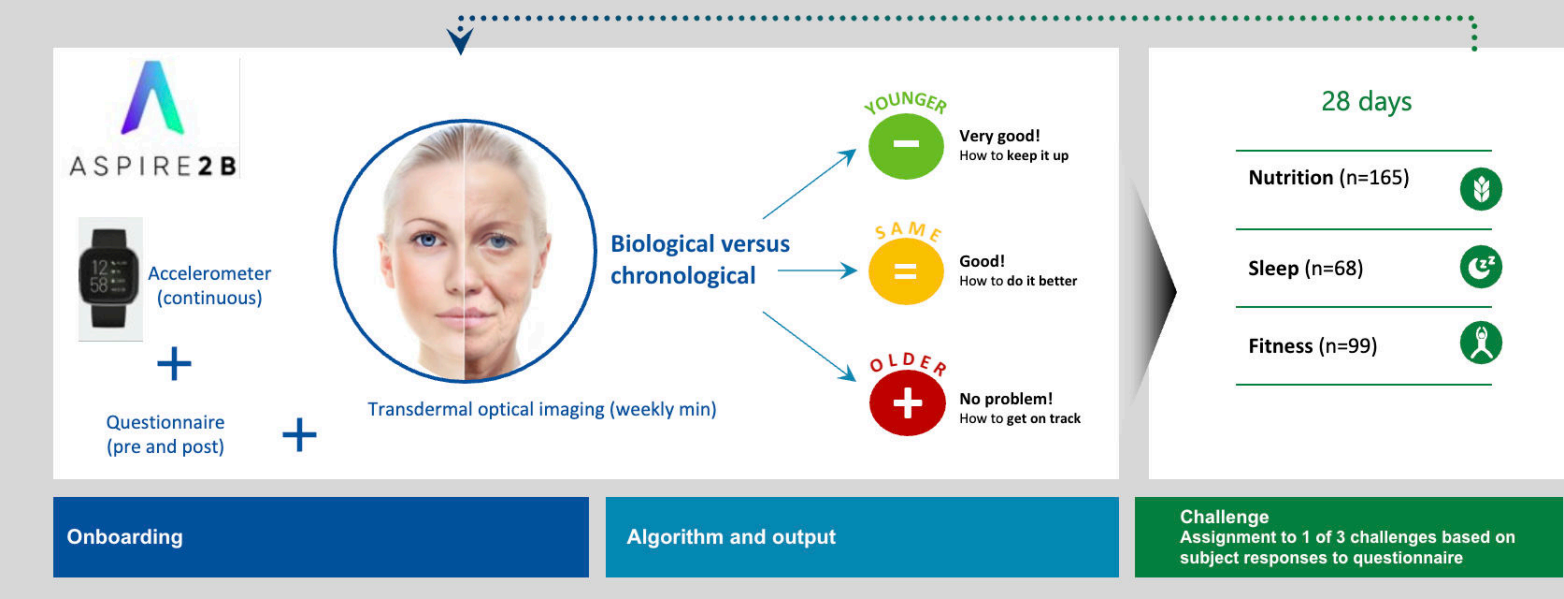
User flow diagram for participants completing a 4-week pilot study using Aspire2B, a personalized wellness app.

Aspire2B, powered by Salus Optima, is a personalized wellness app (Figure 1) that is rooted in evidence-supported techniques that facilitate positive behavioral shifts (eg, improved sleep quality, nutrition, and exercise). After downloading the app, users create a profile (refer to Required Onboarding Questions section), identify their personal wellness goals, and complete face scans that immediately display wellness metrics (ie, biological age, heart rate, blood pressure, and stress index). Biological age score was calculated through the Gero AI algorithm, a machine learning framework [10], and their blood pressure, heart rate, and stress index were estimated using DeepAffex technology (developed by NuraLogix) [13]. To further personalize the experience of Aspire2B, participants were encouraged to answer more questions covering aspects like sleep quality, physical activity, and dietary intake, although these were not mandatory (refer to Optional Onboarding Questions section). From these responses, subjects were assigned to one of three challenges, that are (1) nutrition, (2) movement, (3) sleep based on set criteria (eg, those reporting poor sleep quality were placed in sleep challenge). Those who skipped these questions were prompted to complete them later, with the default challenge assigned as nutrition. Each participant set 28-day goals to guide their progress. Furthermore, Aspire2B streamlines the user experience through integration with Apple Health and Google Fit, enhancing personalized feedback and progress tracking.

#### Educational Content

The app content included a variety of tools to help users work toward their personal wellness goals. Participants could access educational tools at any time, such as exercise videos, articles, tips, and healthy recipes. All articles and tips were grounded in science and supported by peer-reviewed publications (Multimedia Appendix 1).

#### Wellness Tracking

Participants had constant access in the app to a face scan that could provide them with a biological age score, heart rate, blood pressure, and stress index. The DeepAffex Anura face scan technology uses transdermal optimal imaging via photoplethysmography to detect facial blood flow patterns. With a conventional video camera and a cloud-based Affective AI engine, it is possible to predict various health metrics from a scan. The mean difference between the Anura measurement and its paired auscultatory reference measurement has been found to be within acceptable limits [14,15]. Participants could also monitor their step count in the app, which was automatically imported from the user’s device into Aspire2B, and sleep time and quality via self-report (ie, number of hours and sleep quality on a 5-point scale with emojis as anchors). Participants were also able to rate their mood on a 9-point emoji scale (adapted from study by Desmet et al [16]) to monitor changes in mood. Participants were assigned a wellness score, which was generated from participant progress on step count, sleep (time and quality), and mood. Each component was weighted in its contribution to the wellness score and required meeting the expert identified targets (eg, 10,000 steps per day).

#### Notifications and Awards

Aspire2B attempts to take a gamified approach to acknowledge achievements and encourage sustained effort. Digital badges are used to recognize the attainment of subgoals or the unlocking of new levels. To promote user engagement, Aspire2B uses a tailored reminder system that allows users to set or customize reminders. While these features are present, they were not refined or optimized for this trial as the intention of Aspire2B was to serve as a minimum viable product to test the interest in the biological age concept and acceptance of face scan technology.

### Measures

#### Required Onboarding Questions

##### Demographics

Participants were asked demographic questions, such as age, gender, race, and income.

##### BMI

Participants were asked their height and weight to be able to calculate BMI.

##### General Health

Participants were asked how old they felt, to rate their health on a 5-point scale from excellent to poor, and to list how many days their physical health was not good over the last 30 days.

##### Physical Activity

Participants were asked to indicate whether they have engaged in moderate and vigorous physical activities in the past 30 days.

##### Oat Intake

Participants were asked how many servings of oats they ate per week.

##### Mood

Participants were asked to indicate feelings of little energy and little pleasure over the past 2 weeks on a scale from 1 (not at all) to 4 (nearly every day).

### Optional Onboarding Questions

#### Sleep Quality

Participants were asked to rate their sleep quality over the past 7 days on a scale from 0 to 10.

#### Eating Behavior

Participants were asked to indicate how many servings per day they had vegetables, fruit, sugar-sweetened beverages, and whole grains, as well as how many servings they had per week of beans or lentils, fish, nuts, seeds or nut butter, and red or processed meat.

#### Movement

Participants were asked to describe their usual daily activities with respect to sitting, standing, lifting, and stair climbing.

#### Alcohol Consumption

Participants were asked to indicate how many drinks they have in a typical week.

#### Smoking

Participants were asked to indicate if they currently smoke cigarettes and if yes, whether they smoked more than 1 pack per day.

### Optional Offboarding Questions

#### Health Behavior

Participants were asked to repeat questions from the onboarding including body weight, general health, physical activity and movement, mood, sleep, eating behavior including oat intake, and alcohol and cigarette use.

#### Credibility

Participants were asked to respond “yes” or “no” to the question: “Do you find Aspire2B credible?”

#### General Experience

Participants were asked to indicate how likely they would be to recommend Aspire2B to others on a scale of 1 (not recommend) to 10 (strongly recommend).

#### Face Scan Experience

Participants were asked if they liked the face scan experience on a scale of 1 (strongly disagree) to 4 (strongly agree).

#### Face Scan Insights

Participants were asked to answer “yes” or “no” for if they would use the face scan technology to obtain other insights into their health.

### Data Analyses

All data were analyzed using IBM SPSS Statistics (Version 28), JMP Pro Software (Version 17.2) or Jamovi (Version 2.3.21.0). When not specified otherwise, analyses used the full dataset available, with baseline demographic variables (eg, age) derived from enrollment data and engagement metrics analyzed as cumulative measures independent of specific time point. Descriptive statistics were used to summarize and describe the data. Comparisons between Aspire2B participants and the general US population were completed qualitatively using data from the National Health and Nutrition Examination Survey (NHANES) and Behavioral Risk Factor Surveillance System (BRFSS) survey, using the most recently collected data available at the time of analysis (2017‐2018 for NHANES [17] and 2021 for BRFSS [18]). BRFSS was used for comparisons of age group, gender, race or ethnicity, US census region, employment status, education, income, BMI, smoking status, and general health. NHANES was used for alcohol, physical activity, and days of poor physical or mental health. For BRFSS, analysis was restricted to individuals reporting owning a cell phone and who were in the same age range as Aspire2B participants. For NHANES, data on cell phone ownership were not available, but the population was restricted to the same age range. To identify participant characteristics related to engagement, Pearson correlations were conducted. In order to inform future data driven algorithms, additional exploratory general linear models were conducted to identify if the 4-week program led to any lifestyle changes for participants, and whether there was an interaction with age.

## Results

### Participant Characteristics

Participants were aged 20‐65 (mean 40.15, SD 9.59) years. The majority of participants were White females with a bachelor’s degree or above (Table 1). Based on participant responses to onboarding questions, we found that, as compared with the BRFSS [19] or the NHANES [20], participants had similar BMI distributions, but were more likely to consume alcohol frequently. Participants were also more likely to report feeling tired and little pleasure in doing things and were less likely to report “excellent” self-rated health. However, participants were more likely to report engaging in moderate or vigorous physical activities and were less likely to be smokers. At onboarding, participants in each challenge (nutrition, movement, and sleep) were similar regarding height, weight, and BMI, but were statistically different in terms of age, such that those in the sleep challenge had significantly lower chronological age (*P*=.02).

**Table 1.** Demographics for participants who downloaded the Aspire2B app. Participant ages have been grouped in this table for clarity and ease of interpretation, but they are not grouped in the analyses. In addition, demographic data are missing for 16 people.

Characteristics		All participants, n (%)	Nutrition challenge, n (%)	Movement challenge, n (%)	Sleep challenge, n (%)
**Age group (y)**					
	18‐26	19 (5)	7 (1.8)	4 (1)	7 (1.8)
	26‐35	112 (29.3)	43 (11.3)	33 (8.6)	21 (5.5)
	36‐45	142 (37.2)	64 (16.8)	33 (8.6)	31 (8.1)
	46‐55	79 (20.7)	36 (9.4)	22 (5.8)	7 (1.8)
	56‐65	30 (7.9)	15 (3.9)	9 (2.4)	3 (0.8)
**Sex**					
	Male	105 (27.5)	42 (11)	21 (5.5)	21 (5.5)
	Female	277 (72.5)	123 (32.2)	80 (20.9)	48 (12.6)
**Race or ethnicity**					
	White or Caucasian	277 (72.5)	115 (30.1)	74 (19.4)	53 (13.9)
	Black or African American	37 (9.7)	14 (3.7)	9 (2.4)	7 (1.8)
	Hispanic or Latinx	35 (9.2)	17 (4.5)	8 (2.1)	7 (1.8)
	Native American or Alaska Native	1 (0.3)	0 (0)	1 (0.3)	0 (0)
	Multiracial or Biracial	12 (3.1)	7 (1.8)	3 (0.8)	1 (0.3)
	A race or ethnicity not listed here	20 (5.2)	12 (3.1)	6 (1.6)	1 (0.3)
**Employment status**					
	Employed full-time	225 (58.9)	90 (23.6)	59 (15.4)	44 (11.5)
	Employed part-time	41 (10.7)	25 (6.5)	8 (2.1)	4 (1)
	Self-employed	21 (5.5)	3 (0.8)	10 (2.6)	5 (1.3)
	Unemployed	21 (5.5)	11 (2.9)	3 (0.8)	7 (1.8)
	Homemaker	57 (14.9)	29 (7.6)	15 (3.9)	8 (2.1)
	Retired	7 (1.8)	4 (1)	2 (0.5)	0 (0)
	Student	10 (2.6)	3 (0.8)	4 (1)	1 (0.3)
**Education**					
	High-school degree or GED	34 (8.9)	15 (3.9)	6 (1.6)	8 (2.1)
	Some college but no degree	67 (17.5)	28 (7.3)	18 (4.7)	13 (3.4)
	Associate degree (eg, AA and AS)	47 (12.3)	25 (6.5)	11 (2.9)	8 (2.1)
	Bachelor’s degree (eg, BA, BBA, and BS)	164 (42.9)	69 (18.1)	47 (12.3)	28 (7.3)
	Master’s degree (eg, MA, MS, and MEng)	53 (13.9)	22 (5.8)	13 (3.4)	11 (2.9)
	Doctorate (eg, PhD and EdD)	7 (1.8)	3 (0.8)	0 (0)	1 (0.3)
	Professional degree (eg, MD, DDS, and JD)	10 (2.6)	3 (0.8)	6 (1.6)	0 (0)
**Income (US $)**					
	Less than 29,000	22 (5.8)	11 (2.9)	3 (0.8)	6 (1.6)
	30,000-49,999	54 (14.1)	19 (5)	16 (4.2)	11 (2.9)
	50,000-74,999	88 (23)	35 (9.2)	22 (5.8)	21 (5.5)
	75,000-99,999	76 (19.9)	34 (8.9)	24 (6.3)	12 (3.1)
	100,000-150,000	92 (24.1)	41 (10.7)	21 (5.5)	17 (4.5)
	Over 150,000	50 (13.1)	25 (6.5)	15 (3.9)	2 (0.5)

### Acceptability

The majority (111/125, 88.8%) of users who responded to the offboarding questions found Aspire2B credible. In addition, on a scale of 1 to 10, participants would generally recommend Aspire2B to others (mean 6.91, SD 2.45). Only 7.2% (9/125) of users disliked the face scan experience, with 64.8% (81/125) who liked the face scan experience and 83.2% (104/125) said they would use face scan technology for other insights into their health.

### Retention

**Figure 2.**
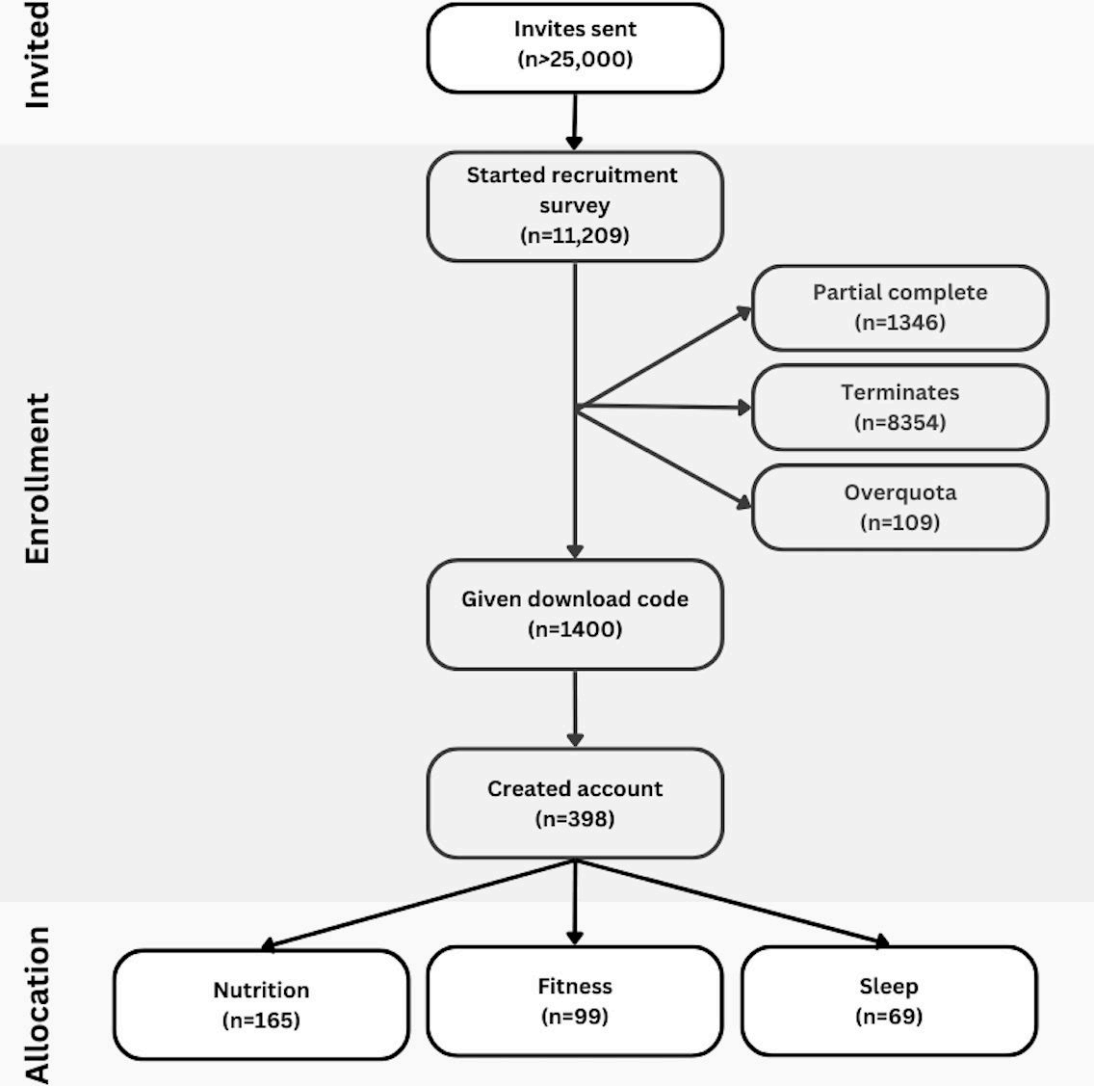
CONSORT (Consolidated Standards of Reporting Trials) flow diagram.

Out of the 398 people who created an account (Figure 2), 85.9% (342/398) completed onboarding and a face scan. Following this, 74.9% (298/398) of users completed the additional survey questions. Notably, interaction with the app was the same during weeks 2 and 4 (final week) of the program (173/398, 43.5%), outperforming industry standards [12] by roughly 3× (Table 2). The majority of participants were placed in the nutrition challenge (n=165), followed by the movement challenge (n=99) and sleep challenge (n=68). Similarly, retention rates were highest for the nutrition intervention (93/165, 56.3%), followed by exercise (49/99, 49.5%) and sleep interventions (27/68, 39.7%), though differences between groups were not significantly different. On average, participants completed 2.1‐2.7 face scans per week, with approximately 7% (24/342) of participants maintaining regular use of face scan technology for 4 weeks.

**Table 2.** Retention rates throughout a 4-week pilot study evaluating acceptability and utility of Aspire2B, a personalized wellness app, as compared with industry standards for wellness apps [12]. Week 2 and 4 interaction for Aspire2B overall percentage engagement were determined by comparing those who interacted with the app during that week with those who created an account. For the individual nutrition, fitness, and sleep challenges, percent engagement was determined by comparing those who interacted with the app during a specific week with those who completed onboarding and were assigned to a challenge. In addition, completing onboarding includes completion of the face scan for Aspire2B.

Enrollment stage	Industry standards, %	Aspire2B overall, %	Nutrition challenge, %	Fitness challenge, %	Sleep challenge, %
Created account	—^a^	398	—^b^	—^b^	—^b^
Completed onboarding	20	86	41	25	17
Week 2 interaction	9	43	60	58	47
Week 4 interaction	4	43	56	49	40
Offboarding completed	—^a^	33	47	38	26

a Not available.

b Not applicable.

### Participant Characteristics Related to Engagement

Age was correlated with engagement across many domains. More specifically, older participants were more likely to log their sleep (*r*_382_=0.21, *P*<.001) and mood (*r*_382_=0.22, *P*<.001), as well as open tips (*r*_382_=0.21, *P*<.001), view their biological age (*r*_382_=0.18, *P*<.001), complete face scans (*r*_382_=0.14, *P*=.005), and finish app challenges (*r*_382_=0.15, *P*=.004).

### Exploratory Analyses

It is noteworthy that we found an interaction between chronological age and biological age as a predictor of the number of challenges completed (*P*=.02) and total workouts (*P*=.04). Interaction probing [21] suggests that for older individuals, a higher discrepancy for biological age (biological>chronological) is predictive of overall challenge completion and total workout number. On the other hand, for younger adults a similar higher discrepancy is related to fewer challenges completed and total workouts. While other engagement metrics are not predicted by this discrepancy, there is potential to encourage behavior change through interest in this concept. Furthermore, biological age and wellness scores did improve for participants who remained engaged over the 4-week program (*P*=.02 and *P*=.001, respectively). There appeared to be greater improvements in wellness scores for older participants (*P*<.001). Lifestyle factors such as oats consumption and sleep quality improved over the 4-week program (*P*<.001).

## Discussion

### Principal Results

Participants who used Aspire2B were 20‐65 years old (with 36‐45 y being the largest group), predominantly White females with a bachelor’s degree or higher. Most participants found Aspire2B credible and were interested in using the app for health insights. Furthermore, the majority of participants completed the onboarding process, including the face scan. As hypothesized, engagement remained relatively high throughout the study. The majority of participants joined the nutrition challenge, which also had the highest retention rates, followed by the movement and sleep challenges. On average, participants completed a face scan multiple times per week, with a small but consistent group maintaining regular use throughout the 4 weeks. Interestingly, age was correlated with engagement, such that older participants were more engaged in the app across many domains.

Retention rates significantly outperformed industry standards [12]. Participants were placed in 1 of 3 challenges based on their needs. Since even modest levels of personalization of programs [22] have shown increased engagement, it is plausible that the personalized challenge impacted overall engagement. This study also revealed high acceptability among users, with the majority finding Aspire2B credible and would recommend it to others. This suggests that users perceive Aspire2B as a trustworthy and credible platform, which is essential for ensuring user engagement and satisfaction [23]. Furthermore, users self-reported improvements in various lifestyle aspects, offering encouragement for the Aspire2B platform and face scan technology as effective nudging tools.

Biological age itself appears to be a construct that participants find engaging and relevant, as evidenced by their interaction with this feature, particularly among older adults. Notably, the objective was not to evaluate the algorithm for accuracy, but rather to test the concept to determine whether it is appealing and effective in influencing behavior. Older participants revisited biological age more frequently. This is useful information for future work as it indicates a subpopulation of individuals for whom biological age may be salient and may be receptive to setting behavioral goals linked to biological age. For these individuals, a discrepancy between biological age and chronological age may also be motivating for engaging in health behaviors. Importantly, technology (digital application and face scan technology) was found acceptable for older users, offering a viable solution to nudge individuals toward better lifestyle choices.

In addition, patterns were found regarding predictors of user engagement. Higher chronological age correlated with greater engagement across various domains. Notably, those in the sleep arm had significantly lower chronological age, which may explain the higher attrition rate. Older users were more likely to log their sleep and mood, as well as open tips, view their biological age, complete face scans, and finish app challenges. It is unclear whether these behaviors are linked to attempts to manage biological age directly or whether older users are more likely to engage with Aspire2B generally, a concept requiring further testing.

### Limitations and Future Directions

This study is not without limitations. Employees of consumer package goods companies were not excluded from the study. Although it was not openly disclosed that the app was developed by a multinational food and beverage corporation, this information was available in the terms and conditions of the app, which may have introduced bias into the study results. In addition, this study was US-specific and may not generalize to global apps. Future investigation into the feasibility and effectiveness of Aspire2B in a global context, considering cultural and regional variations, is necessary. Furthermore, selection bias may be present, as participants who opted to participate in this study may differ systematically from those who declined, but we cannot assess the extent of this bias since we do not have data from nonparticipants. Markedly, the wellness app in this study serves as a rudimentary platform for hypothesis testing and may be used to improve engagement required in features for a commercial launch.

It may also be beneficial to investigate the impact of increased personalization through the integration of machine learning techniques to determine if they can lead to higher and long-term user engagement. Further personalization by segmenting consumers based on their values and behaviors may also offer increased tailored experiences that improve overall success rates. There may also be interesting differences in user engagement and retention between apps that offer a continuous wellness program and those that follow a finite trial mode, such as the study completed here. Finally, the addition of more features, such as more physiological results revealed through face scan, may also increase user acquisition and retention. Future studies may also benefit from trying to tease out if the face scan technology is the draw or if high engagement was due to attempting to improve one’s biological age through the set program. These future directions can help refine the strategies used in wellness apps, ultimately promoting long-term engagement and effectively fostering healthier behaviors in users.

### Conclusions

Overall, this study highlights the potential of Aspire2B as a credible and engaging wellness app, particularly for older adults, as engagement levels were positively correlated with age across various features. This study provides a foundation for further exploration of personalized challenges, biological age feedback, and user-friendly face scan technology to drive engagement, and therefore, potentially improvements in lifestyle behavior. Importantly, the high retention rates indicate a promising model for future wellness technologies.

## Supplementary material

10.2196/63471 Multimedia Appendix 1 Application educational content.

## Abbreviations

BRFSS: Behavioral Risk Factor Surveillance System
NHANES: National Health and Nutrition Examination Survey
