# Supplementary material for: Evaluating the Acceptability and Utility of a Personalized Wellness App (Aspire2B) Using AI-Enabled Digital Biomarkers: Engagement Enhancement Pilot Study
Source: JMIR Form Res. 2025 May 14;9:e63471. doi: 10.2196/63471 (PMC12094527; doi:10.2196/63471)
Supplement: Multimedia Appendix 1 [file formative-v9-e63471-s001.docx]

1. Consensus Conference Panel, Watson NF, Badr MS, et al. Joint Consensus Statement of the American Academy of Sleep Medicine and Sleep Research Society on the Recommended Amount of Sleep for a Healthy Adult: Methodology and Discussion. Sleep. 2015;38(8):1161-1183. doi:10.5665/sleep.4886

2. Shammas MA. Telomeres, lifestyle, cancer, and aging. Current Opinion in Clinical Nutrition & Metabolic Care. 2011;14(1):28. doi:10.1097/MCO.0b013e32834121b1

3. How Much Sleep Do You Need? Sleep Foundation. March 9, 2021. Accessed October 8, 2024. https://www.sleepfoundation.org/how-sleep-works/how-much-sleep-do-we-really-need

4. Spira AP, Chen-Edinboro LP, Wu MN, Yaffe K. Impact of sleep on the risk of cognitive decline and dementia. Current Opinion in Psychiatry. 2014;27(6):478. doi:10.1097/YCO.0000000000000106

5. Best Temperature for Sleep. Sleep Foundation. October 29, 2020. Accessed October 8, 2024. https://www.sleepfoundation.org/bedroom-environment/best-temperature-for-sleep

6. Okamoto-Mizuno K, Mizuno K. Effects of thermal environment on sleep and circadian rhythm. Journal of Physiological Anthropology. 2012;31(1):14. doi:10.1186/1880-6805-31-14

7. Haghayegh S, Khoshnevis S, Smolensky MH, Diller KR, Castriotta RJ. Before-bedtime passive body heating by warm shower or bath to improve sleep: A systematic review and meta-analysis. Sleep Medicine Reviews. 2019;46:124-135. doi:10.1016/j.smrv.2019.04.008

8. Kredlow MA, Capozzoli MC, Hearon BA, Calkins AW, Otto MW. The effects of physical activity on sleep: a meta-analytic review. J Behav Med. 2015;38(3):427-449. doi:10.1007/s10865-015-9617-6

9. Pengpid S, Peltzer K. Sedentary Behaviour, Physical Activity and Life Satisfaction, Happiness and Perceived Health Status in University Students from 24 Countries. International Journal of Environmental Research and Public Health. 2019;16(12):2084. doi:10.3390/ijerph16122084

10. Caffeine and Sleep. Sleep Foundation. April 17, 2009. Accessed October 8, 2024. https://www.sleepfoundation.org/nutrition/caffeine-and-sleep

11. Wahl S, Engelhardt M, Schaupp P, Lappe C, Ivanov IV. The inner clock—Blue light sets the human rhythm. Journal of Biophotonics. 2019;12(12):e201900102. doi:10.1002/jbio.201900102

12. Masters A, Pandi-Perumal SR, Seixas A, Girardin JL, McFarlane SI. Melatonin, the Hormone of Darkness: From Sleep Promotion to Ebola Treatment. Brain Disord Ther. 2014;4(1):1000151. doi:10.4172/2168-975X.1000151

13. How Blue Light Affects Kids’ Sleep. Sleep Foundation. September 24, 2020. Accessed October 8, 2024. https://www.sleepfoundation.org/children-and-sleep/how-blue-light-affects-kids-sleep

14. Disrupted sleep in one’s 50s, 60s raises risk of Alzheimer’s disease. ScienceDaily. Accessed October 8, 2024. https://www.sciencedaily.com/releases/2019/06/190627114105.htm

15. Teo JX, Davila S, Yang C, et al. Digital phenotyping by consumer wearables identifies sleep-associated markers of cardiovascular disease risk and biological aging. Commun Biol. 2019;2(1):1-10. doi:10.1038/s42003-019-0605-1

16. Tosh SM. Review of human studies investigating the post-prandial blood-glucose lowering ability of oat and barley food products. Eur J Clin Nutr. 2013;67(4):310-317. doi:10.1038/ejcn.2013.25

17. Haminiuk CWI, Maciel GM, Plata-Oviedo MSV, Peralta RM. Phenolic compounds in fruits – an overview. International Journal of Food Science & Technology. 2012;47(10):2023-2044. doi:10.1111/j.1365-2621.2012.03067.x

18. Tabart J, Franck T, Kevers C, et al. Antioxidant and anti-inflammatory activities of Ribes nigrum extracts. Food Chemistry. 2012;131(4):1116-1122. doi:10.1016/j.foodchem.2011.09.076

19. Steinberg FM, Batchelor-Murphy MK, Young HM. Supporting Family Caregivers: No Longer Home Alone: Eating for Healthy Aging. AJN The American Journal of Nursing. 2019;119(11):43. doi:10.1097/01.NAJ.0000605360.22194.1f

20. Chu Y. Oats Nutrition and Technology. John Wiley & Sons; 2013. Accessed October 7, 2024. https://books.google.com/books?hl=en&lr=&id=jVnSAQAAQBAJ&oi=fnd&pg=PA1951&dq=Oats+Nutrition+and+Technology&ots=rICp2Td_Se&sig=KdgkwYtP6jkmIkM3YAutc2WrC-0

21. Sang S, Chu Y. Whole grain oats, more than just a fiber: Role of unique phytochemicals. Molecular Nutrition & Food Research. 2017;61(7):1600715. doi:10.1002/mnfr.201600715

22. Han KT, Kim DW, Kim SJ, Kim SJ. Biological Age Is Associated with the Active Use of Nutrition Data. International Journal of Environmental Research and Public Health. 2018;15(11):2431. doi:10.3390/ijerph15112431

23. Rasane P, Jha A, Sabikhi L, Kumar A, Unnikrishnan VS. Nutritional advantages of oats and opportunities for its processing as value added foods - a review. J Food Sci Technol. 2015;52(2):662-675. doi:10.1007/s13197-013-1072-1

24. Chen O, Mah E, Dioum E, et al. The Role of Oat Nutrients in the Immune System: A Narrative Review. Nutrients. 2021;13(4):1048. doi:10.3390/nu13041048

25. Calder PC, Carding SR, Christopher G, Kuh D, Langley-Evans SC, McNulty H. A holistic approach to healthy ageing: how can people live longer, healthier lives? Journal of Human Nutrition and Dietetics. 2018;31(4):439-450. doi:10.1111/jhn.12566

26. Steffen LM, Kroenke CH, Yu X, et al. Associations of plant food, dairy product, and meat intakes with 15-y incidence of elevated blood pressure in young black and white adults: the Coronary Artery Risk Development in Young Adults (CARDIA) Study2. The American Journal of Clinical Nutrition. 2005;82(6):1169-1177. doi:10.1093/ajcn/82.6.1169

27. Willcox DC, Willcox BJ, Todoriki H, Suzuki M. The Okinawan Diet: Health Implications of a Low-Calorie, Nutrient-Dense, Antioxidant-Rich Dietary Pattern Low in Glycemic Load. Journal of the American College of Nutrition. 2009;28(sup4):500S-516S. doi:10.1080/07315724.2009.10718117

28. Whitehead A, Beck EJ, Tosh S, Wolever TM. Cholesterol-lowering effects of oat β-glucan: a meta-analysis of randomized controlled trials1234. The American Journal of Clinical Nutrition. 2014;100(6):1413-1421. doi:10.3945/ajcn.114.086108

29. Bazzano LA, He J, Ogden LG, Loria CM, Whelton PK. Dietary Fiber Intake and Reduced Risk of Coronary Heart Disease in US Men and Women: The National Health and Nutrition Examination Survey I Epidemiologic Follow-up Study. Archives of Internal Medicine. 2003;163(16):1897-1904. doi:10.1001/archinte.163.16.1897

30. Davy BM, Davy KP, Ho RC, Beske SD, Davrath LR, Melby CL. High-fiber oat cereal compared with wheat cereal consumption favorably alters LDL-cholesterol subclass and particle numbers in middle-aged and older men123. The American Journal of Clinical Nutrition. 2002;76(2):351-358. doi:10.1093/ajcn/76.2.351

31. Behall KM, Scholfield DJ, Hallfrisch J. Comparison of Hormone and Glucose Responses of Overweight Women to Barley and Oats. Journal of the American College of Nutrition. 2005;24(3):182-188. doi:10.1080/07315724.2005.10719464

32. Hou Q, Li Y, Li L, et al. The Metabolic Effects of Oats Intake in Patients with Type 2 Diabetes: A Systematic Review and Meta-Analysis. Nutrients. 2015;7(12):10369-10387. doi:10.3390/nu7125536

33. Gibala MJ, Little JP, MacDonald MJ, Hawley JA. Physiological adaptations to low-volume, high-intensity interval training in health and disease. The Journal of Physiology. 2012;590(5):1077-1084. doi:10.1113/jphysiol.2011.224725

34. Nakamura E, Moritani T, Kanetaka A. Biological age versus physical fitness age. Europ J Appl Physiol. 1989;58(7):778-785. doi:10.1007/BF00637391

35. Nakamura E, Moritani T, Kanetaka A. Further evaluation of physical fitness age versus physiological age in women. Eur J Appl Physiol. 1998;78(3):195-200. doi:10.1007/s004210050407

36. Nakamura E, Moritani T, Kanetaka A. Effects of habitual physical exercise on physiological age in men aged 20–85 years as estimated using principal component analysis. Europ J Appl Physiol. 1996;73(5):410-418. doi:10.1007/BF00334417

37. Tucker LA. Physical activity and telomere length in U.S. men and women: An NHANES investigation. Preventive Medicine. 2017;100:145-151. doi:10.1016/j.ypmed.2017.04.027

38. Chodzko-Zajko WJ, Proctor DN, Fiatarone Singh MA, et al. Exercise and Physical Activity for Older Adults. Medicine & Science in Sports & Exercise. 2009;41(7):1510. doi:10.1249/MSS.0b013e3181a0c95c

39. Stork MJ, Banfield LE, Gibala MJ, Martin Ginis KA. A scoping review of the psychological responses to interval exercise: is interval exercise a viable alternative to traditional exercise? Health Psychology Review. 2017;11(4):324-344. doi:10.1080/17437199.2017.1326011

40. Fitness In-Depth. Mayo Clinic. Accessed October 7, 2024. https://www.mayoclinic.org/healthy-lifestyle/fitness/basics/fitness-basics/hlv-20049447

41. Current Guidelines | health.gov. Accessed October 7, 2024. https://health.gov/our-work/nutrition-physical-activity/physical-activity-guidelines/current-guidelines

42. Gibala MJ, Gillen JB, Percival ME. Physiological and Health-Related Adaptations to Low-Volume Interval Training: Influences of Nutrition and Sex. Sports Med. 2014;44(2):127-137. doi:10.1007/s40279-014-0259-6

43. Gries KJ, Raue U, Perkins RK, et al. Cardiovascular and skeletal muscle health with lifelong exercise. Journal of Applied Physiology. 2018;125(5):1636-1645. doi:10.1152/japplphysiol.00174.2018

44. Gibala M, Shulgan C. The One-Minute Workout: Science Shows a Way to Get Fit That’s Smarter, Faster, Shorter. Penguin; 2017. Accessed October 7, 2024. https://books.google.com/books?hl=en&lr=&id=dvbbDQAAQBAJ&oi=fnd&pg=PA1&dq=The+One-Minute+Workout:+Science+Shows+a+Way+to+Get+Fit+That%27s+Smarter,+Faster,+Shorter+&ots=f0XRfRRdb4&sig=rIc1uFe5Av1q30Zs6bmfj23hr_o

45. Lavin KM, Roberts BM, Fry CS, Moro T, Rasmussen BB, Bamman MM. The Importance of Resistance Exercise Training to Combat Neuromuscular Aging. Physiology. 2019;34(2):112-122. doi:10.1152/physiol.00044.2018

46. Morawin B, Tylutka A, Chmielowiec J, Zembron-Lacny A. Circulating Mediators of Apoptosis and Inflammation in Aging; Physical Exercise Intervention. International Journal of Environmental Research and Public Health. 2021;18(6):3165. doi:10.3390/ijerph18063165

47. Harb SC, Cremer PC, Wu Y, et al. Estimated age based on exercise stress testing performance outperforms chronological age in predicting mortality. European Journal of Preventive Cardiology. 2021;28(12):1295-1302. doi:10.1177/2047487319826400

48. CDC. Chronic Disease. Chronic Disease. May 24, 2024. Accessed October 7, 2024. https://www.cdc.gov/chronic-disease/index.html

49. Levine ME, Crimmins EM. Is 60 the New 50? Examining Changes in Biological Age Over the Past Two Decades. Demography. 2018;55(2):387-402. doi:10.1007/s13524-017-0644-5

50. Wickramasinghe K, Mathers JC, Wopereis S, Marsman DS, Griffiths JC. From lifespan to healthspan: the role of nutrition in healthy ageing. Journal of Nutritional Science. 2020;9:e33. doi:10.1017/jns.2020.26

51. Kennedy BK, Berger SL, Brunet A, et al. Geroscience: Linking Aging to Chronic Disease. Cell. 2014;159(4):709-713. doi:10.1016/j.cell.2014.10.039

52. Karasik D, Demissie S, Cupples LA, Kiel DP. Disentangling the Genetic Determinants of Human Aging: Biological Age as an Alternative to the Use of Survival Measures. The Journals of Gerontology: Series A. 2005;60(5):574-587. doi:10.1093/gerona/60.5.574

53. Daytime sleepiness may be linked to genetic markers for longevity. Inverse. April 28, 2021. Accessed October 3, 2024. https://www.inverse.com/mind-body/daytime-sleepiness-may-be-aging-you

54. Kim S, Jazwinski SM. Quantitative measures of healthy aging and biological age. Healthy Aging Res. 2015;4:26. doi:10.12715/har.2015.4.26

55. Rowe JW, Kahn RL. Successful aging. The gerontologist. 1997;37(4):433-440.

56. Avery-Duke S. Effects of faster aging show up by midlife. Futurity. March 23, 2021. Accessed October 3, 2024. https://www.futurity.org/aging-dementia-frailty-signs-2536772/

57. Furman D, Campisi J, Verdin E, et al. Chronic inflammation in the etiology of disease across the life span. Nat Med. 2019;25(12):1822-1832. doi:10.1038/s41591-019-0675-0
